# Finely Designing Dicarboxylic Acid-Based Protic Ionic Liquids System for Tailoring Lignin Structure via Demethylation Strategy

**DOI:** 10.3390/molecules30112445

**Published:** 2025-06-03

**Authors:** Cheng Li, Xinyu Xiao, Qizhen Luo, Wanting Zhao, Wenzhe Xiao, Ling-Ping Xiao, Yao Tong, Shangru Zhai, Jian Sun

**Affiliations:** 1 Liaoning Key Lab of Lignocellulose Chemistry and BioMaterials, Liaoning Collaborative Innovation Center for Lignocellulosic Biorefinery, School of Light Industry and Chemical Engineering, Dalian Polytechnic University, Dalian 116034, China; licheng0227@yeah.net (C.L.); xiaoxinyu96312@foxmail.com (X.X.); lpxiao@dlpu.edu.cn (L.-P.X.); tongyao@dlpu.edu.cn (Y.T.); 2 Key Laboratory of Molecular Medicine and Biotherapy, Ministry of Industry and Information Technology, School of Life Sciences, Beijing Institute of Technology, Beijing 100081, China; 13237127067@163.com (Q.L.); wanting22@126.com (W.Z.); 18846829392@163.com (W.X.); 3 Beijing Engineering Research Center of Cellulose and Its Derivatives, Advanced Research Institute of Multidisciplinary Sciences, Beijing Institute of Technology, Beijing 100081, China; 4 School of Environmental and Nature Resources, Zhejiang University of Science and Technology, Hangzhou 310023, China

**Keywords:** lignin, demethylation strategy, ionic liquids, polyphenol, value-added utilization

## Abstract

As one kind of renewable aromatic polymer, lignin is severely underused due to its chemical recalcitrance. Lignin can endure demethylation modification to improve its activation by releasing more active functional groups. However, the process suffers from expensive, corrosive, and toxic issues by employing halogen-containing reagents, which has become an obstacle to industrial applications. Herein, a series of dicarboxylic acid-based protic ionic liquids (DAPILs) systems composed of ethanolamine and dibasic organic acids (e.g., aspartic acid (Asp), glutamic acid (Glu), succinic acid (SA), and glutaric acid (GA)) with 1~2:1 stoichiometric ratio, have been finely designed for the demethylation of industrial lignin. With [EOA][GA] treatment, the polyphenol content in lignin was favorably increased beyond 1.58 times. The structural tailoring and variation were fully characterized by 2D HSQC and ^1^H NMR. The analysis results indicated that, with the increase of phenolic hydroxyl content in lignin, the β-*O*-4′ bond was broken and the content of structural units (S, G) and the S/G ratio of lignin decreased accordingly. After the treatment, the used IL and tailored lignin can be recovered over 95%. This novel, halogen-free and environmentally friendly lignin-cutting strategy not only opens avenues for high-value utilization of lignin but also expands the field of application of dicarboxylic acid-based protic ionic liquids.

## 1. Introduction

Lignocellulosic biomass is a renewable, biodegradable, non-toxic, and abundant natural energy source, with the potential to replace fossil fuels in the future [1]. Lignin, a key component of lignocellulosic biomass, is a major global source of aromatic compounds and holds significant potential for producing a variety of chemical products [2], including pharmaceuticals [3], adhesive [4], antioxidants [5], and anti-inflammatory agents [5]. Lignin consists of three structural units: syringyl (S), guaiacyl (G), and p-hydroxyphenyl (H) [6]. The G-type lignin structural unit features a methoxy group at the C3 position. In contrast, the S-type lignin structural unit possesses two methoxy groups, located at the C3 and C5 positions, respectively. Conversely, the H-type lignin structural unit lacks any methoxy groups. Lignin is primarily derived from waste pulp in the paper industry, with an estimated annual global production of approximately 50 million tons [7]. However, only 2% of lignin waste is currently utilized for high-value applications, such as modifications to enhance utilization, while 98% is used for lower-value purposes, such as combustion for energy generation [8]. Therefore, research on the modification of lignin to facilitate its high-value, value-added utilization is of practical importance.

Modifying lignin is also challenging due to its complex aromatic structure, low reactivity, and poor solubility. The molecular structure of lignin contains several reactive functional groups, such as aromatic rings, phenolic hydroxyls (Ph-OH), carbonyls, and methoxyls [9]. These groups enable a variety of chemical reactions, including halogenation, phenolization [10,11], graft copolymerization [12], alkylation and dealkylation [13], acylation [14], amination [12,15], esterification [16], and hydrolysis [17]. Although these methods can improve lignin’s reactivity, they are constrained by complex modification conditions. Therefore, developing novel strategies that can operate under mild conditions in a short time and are easy to manipulate is an urgent need. Lignin contains numerous methoxy groups and β-*O*-4′ bonds in its structure. The depolymerization of the β-*O*-4′ bond has been reported in the literature, such as the pyrolysis of the β-*O*-4′ bond to produce phenolic compounds [18], and electrocatalytic oxidative degradation of the β-*O*-4′ bond to yield carboxylic acids, aromatic aldehydes, and other high-value products [19]. During demethylation, a nucleophilic attack on the methoxy group may lead to the loss of methyl groups and the release of free Ph-OH [20]. Simultaneously, the cleavage of the β-*O*-4′ bond also produces additional Ph-OH, significantly increasing the Ph-OH content in lignin [21].

The complex structure of industrial lignin, coupled with the consumption of various functional groups during processing, leads to the breaking of ether bonds and a reduction in reactivity, thereby enhancing lignin’s chemical stability. Traditional demethylation methods include Lewis acid catalysis [22,23], redox reactions (e.g., bacterial and fungal processes) [24], thermal cleavage [25], and microwave heating [26]. However, most of these methods necessitate harsh conditions, such as high pressure, elevated temperatures, metal catalysts, or halogenated acids, which can lead to equipment corrosion. While microwave heating has shown effectiveness in lignin modification, its industrialization is hindered by the high cost of the required equipment. Furthermore, the demethylation process yields different products under varying catalytic conditions. According to Mei et al., in the presence of a cocatalyst, methoxyl groups can react with CO and water to produce acetic acid, achieving a high conversion rate [27]. The demethylation of lignin using sodium ethyl mercaptan (EtSNa) as a nucleophile produces methyl ethyl sulfide as a by-product, which has a pungent odor [28]. Under different conditions, lignin also generates various condensation products. For instance, under acidic conditions, lignin undergoes C-C bond formation through a possible condensation mechanism, and the acid hydrolysis of β-*O*-4′ bonds also produces phenylacetaldehyde [29].

Ionic liquids (ILs) are organic molten salts that exist as free ions in the liquid state at or near room temperature. ILs are frequently used as green solvents for catalysis, organic synthesis, and material preparation, owing to their unique physicochemical properties, such as non-flammability, non-volatility, good electrical conductivity, and recyclability [30]. Generally, ILs can be classified into protic, aprotic, zwitterionic, and polymeric types, which have found wide applications in biomass pretreatment [31], battery electrolytes [32], additives, membranes [33], and other industrial applications. In biomass pretreatment, ILs can not only act as solvents to dissolve biomass at appropriate temperatures, but also act as reactants to react with biomass, thereby achieving the effect of biomass modification [34,35,36]. Sun et al. employed a protic ionic liquid, ethanolamine acetate ([EOA][OAc]), for the one-pot production of cellulosic ethanol, achieving ethanol yields of up to 70% of the theoretical maximum. The IL, [EOA][OAc], synthesized through a simple acid-based reaction, is biocompatible and cost-effective for bioethanol production under optimal pH and high loading conditions [37]. More recently, the same group demonstrated the use of prepared lignin polyphenols as tanning agents for chrome-free eco-leather production, aiming to address issues in the leather industry, such as the need for harsh conditions and toxic reagents [38,39]. Despite the successes of [EOA][OAc], the volatile nature of acetic acid can lead to changes in the composition of the IL system, affecting the demethylation performance during recycling. Furthermore, the IL contains only one active carboxylic acid site, so the potential of protic ionic liquid in lignin demethylation requires further exploration. Therefore, in pursuit of a more sustainable treatment process, there is a growing demand to design IL systems with multiple carboxylic acid active sites to efficiently tailor lignin structures.

**Scheme 1:**
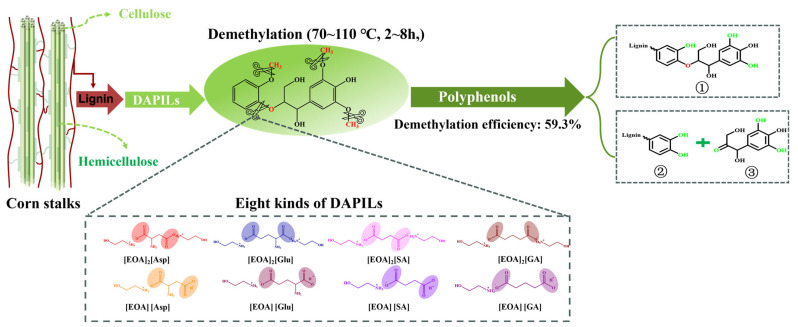
Demethylation processing of lignin by DAPILs.

Inspired by the above viewpoints, a continuous endeavor is undertaken to develop and explore new strategies toward lignin demethylation for the high-value-added utilization. Herein, eight dicarboxylic acid-based protic ionic liquids (DAPILs) were prepared at different stoichiometric ratios using ethanolamine (EOA) as the positive ion and aspartic acid (Asp), glutamic acid (Glu), succinic acid (SA), and glutaric acid (GA) as the anions. Lignin was demethylated using these different DAPILs (Scheme 1), respectively. As shown in Scheme 1, this scheme contains two different approaches, namely the demethylation of lignin (①), and the demethylation accompanied by the breaking of ether bonds simultaneously (②, ③). The lignin samples obtained from different DAPILs treatments were systematically characterized by Fourier Transform Infrared Spectroscopy (FTIR), Ultraviolet Spectroscopy (UV), Gel Permeation Chromatography (GPC), two-dimensional Heteronuclear Single Quantum Coherence (2D HSQC), and Nuclear Magnetic Resonance (NMR) to reveal the structural changes of lignin.

## 2. Results and Discussions

### 2.1. Preparation and Characterization of DAPILs

**Figure 1:**
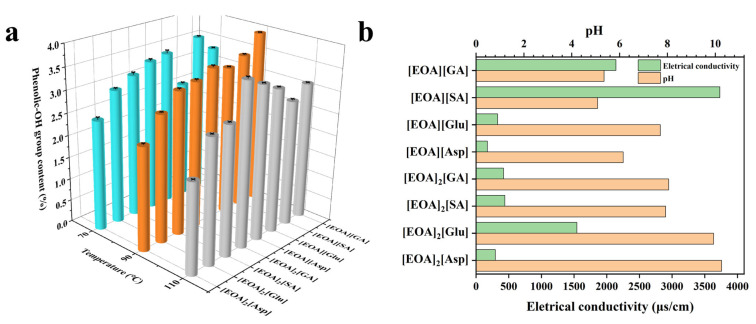
(**a**) Effect of eight DAPILs on phenolic hydroxyl content in al-modified lignin at different temperatures (conditions: 2 h, DAPILs to lignin AL mass ratio is 4:1). (**b**) Conductivity and pH of DAPILs.

To screen the most effective DAPILs from the eight prepared candidates of ILs, the purified alkali lignin (AL) was selected as the substrate. The weight ratio of AL to DAPIL was 1/4. To lower the viscosity of the system, 0.5 mL of water was added, and the mixture was pretreated for 2 h. At the end of the experiment, the contents of Ph-OH in the pretreated lignin (PAL) were determined using UV spectrophotometry. To some extent, the newly formed Ph-OH group in PAL represented the impact on the demethylation of AL. Figure 1 shows DAPILs and the basic tests performed on their performance, where each of the eight DAPILs is shown in Figures S1 and S2, which show the structural formula for the eight DAPILs. For example, a DAPIL with a 2:1 stoichiometric ratio of EOA to Asp is referred to as [EOA]_2_[Asp], and a DAPIL with a 1:1 stoichiometric ratio is referred to as [EOA][Asp]. Figure 1b illustrates the pH and conductivity of the DAPILs, indicating that the pH is higher when the molar ratio of ethanolamine to dibasic acid is 2:1. Given that Ph-OH tends to be weakly acidic, alkaline conditions are not conducive to the generation of PhOH. As shown in Figure 1a, the PHC produced by DAPIL-pretreated lignin under identical conditions at a molar ratio of 1:1 was slightly higher than that at a molar ratio of 2:1. The conductivity, which influenced the transfer of active protons, reached its maximum at [EOA][GA] (Figure 1b). In summary, when the pH is weakly acidic, the conductivity of [EOA][GA] and [EOA][SA] are at large values.

### 2.2. Demethylation and Structural Characterization of Lignin

Experiments on the demethylation effects of lignin at different temperatures (i.e., 70, 90, and 110 °C) were carried out on the eight DAPILs for 2 h. As depicted in Figure 1a, the demethylation effects of all eight ILs were better at 90 °C, which was attributed to the nucleophilic attack of the anions of the ILs on the methoxyl group in the lignin to demethylate the lignin and then transformed to free Ph-OH. In contrast, demethylation was less effective at 70 °C, which was probably since lower temperatures were not favorable for nucleophilic reactions. But at higher temperatures, the simultaneous condensation reaction of lignin demethylation increases, resulting in a decrease in Ph-OH. Since Ph-OH was weakly acidic, more acidic conditions favored demethylation. Whereas the reaction system was more acidic when the EOA content was low, DAPILs with a stoichiometric ratio of 1:1 were therefore more favorable for lignin demethylation.

**Figure 2:**
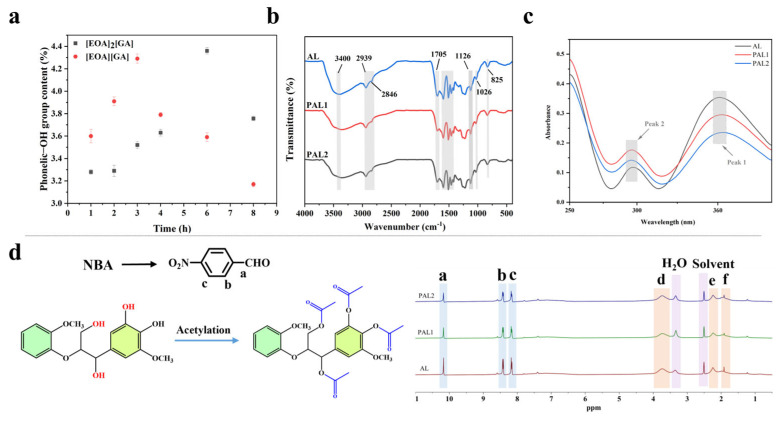
(**a**) Effect of DAPILs with different stoichiometric ratios on AL modification at the same temperature (90 °C) for different times. (**b**) Infrared spectra of AL before and after modification (PAL1: [EOA][GA], 90 °C, 3 h; PAL2: [EOA]_2_[GA], 90 °C, 6 h). (**c**) UV spectra before and after lignin modification (AL, PAL1, PAL2). (**d**) ^1^H NMR after acetylation of AL, PAL1 and PAL2.

By summarizing the demethylation effects of eight DAPILs reacted under different reaction conditions for 2 h, [EOA][GA] reached a higher demethylation effect at 90 °C for 2 h, and the PHC was determined to be 3.91. Therefore, [EOA][GA] was chosen as the main DAPIL to continue the experiment, and [EOA]_2_[GA] DAPIL was used as a control experiment. The two DAPILs were subjected to different reaction times (e.g., 1, 2, 3, 4, 6, and 8 h) on AL at 90 °C. As depicted in Figure 2a, after treatment with the two DAPILs, the content of PHCs in AL increased. Specifically, when AL was modified by [EOA][GA], its PHC reached the maximum value of 4.29 at 3 h and then began to decrease, while that of AL modified by [EOA]_2_[GA] could reach a maximum value of 4.36 at 6 h.

To verify whether there was a change in the functional groups of lignin before and after modification, FT-IR characterizations were conducted (Figure 2b). The peaks at 1026 cm^−1^ and 825 cm^−1^ indicated that the AL used contains H, G, and S structural units, whereas the peaks at 1601 cm^−1^, 1501 cm^−1^ and 1442 cm^−1^ represent the vibration of the aromatic backbone in the lignin [40]. The slight change of the peak at 1126 cm^−1^ indicates that the aromatic ring of lignin is modified. Moreover, the breakage of the ester bonds may also generate Ph-OH. The peak at 1705 cm^−1^ represents the stretching vibration of the lignin carbonyl group [41]. The peaks at 2939 and 2846 cm^−1^ are the C-H stretching vibrations of methyl (-CH_3_) and methylene (-CH_2_) [42], respectively. It was observed that both those peaks were slightly reduced after AL modification, which also indicated the demethylating effect of [EOA][GA] on AL. The larger peak at 3400 cm^−1^ is a phenol/aliphatic hydroxyl (-OH) stretching vibration [43]. From the results of the FTIR spectra, we can see that different ratios of [EOA][GA] have a role in the demethylation process of AL, and the basic skeleton of lignin remains unchanged after the modification.

The demethylation effect of different ratios of DAPILs on AL was reflected to some extent by the calculation of PHC using UV differential absorption method. As shown in Figure 2c, the UV absorption spectra of Ph-OH of lignin were shown at 250~400 nm, where the two characteristic absorption peaks of Ph-OH were displayed at 300 and 360 nm, respectively. The peak intensity was proportional to the concentration of lignin dissolved in dioxane-aqueous solution at 360 nm (i.e., AL: 10.5 mg, PAL1: 7.5 mg, and PAL2: 5.9 mg, respectively). Although the weighing of AL was greater than that of PAL1 and PAL2, the PHC content increased from 2.75% for AL to 4.29% and 4.36%, an increase of 56% and 58%, respectively. All these data also indicated that the two DAPILs underwent different degrees of demethylation of AL.

The ^1^H NMR spectra of acetylated Al, PAL1 and PAL2 are shown in Figure 2d. The proton signal peak at a peak represents the hydrogen proton displacement of aldehyde in the internal standard *p*-nitrobenzaldehyde (NBA) molecule, a and peaks b and c represent the chemical displacement of hydrogen protons on the benzene ring of the internal standard NBA, respectively. The hydrogen proton signal at 3.82 ppm is the lignin methoxyl signal peak (d peak), and the hydrogen proton signal peaks at 2.24 ppm (e peak) and 1.90 ppm (f peak) represent aromatic and aliphatic protons in the acetyl group [44]. The results of calculating the methoxy content and PHC based on the internal standard NBA content and signal peaks are summarized in Table 1. After demethylation, the methoxy content decreased from 1.11 mmol/g for AL to 0.45 mmol/g for PAL1 and 0.61 mmol/g for PAL2, respectively. However, the PHC increased from 0.50 mmol/g for AL to 0.58 mmol/g for PAL1 and 0.61 mmol/g for PAL2, respectively. Compared with PAL2, PAL1 has a higher methoxy removal rate and a lower PHC, which may be due to the acidic environment of PAL1 leading to a small part of Ph-OH condensation [45].

Elemental analysis was performed on AL, PAL1, and PAL2, as shown in Table S1. The fact that there was some reduction in carbon content after lignin demethylation treatment indicated the demethylation effect [46]. On the contrary, the small amount of elemental N in the unreacted lignin was due to the fact that corn stover belongs to the gramineous family of plants, which consisted of carbohydrates and a small number of proteins. The increase in N content in modified lignin may be related to the trace residue of DAPIL. This is because we observed extremely tiny peaks at 1651 cm^−1^ and 864 cm^−1^ in Figure 2b, which respectively represent the vibration peaks of N-H and -NH_2_ [47]. Furthermore, in Figure 2b, we observed the characteristic peak of C-N at 1365 cm^−1^ [48], which indicates that at 90 °C, with the increase of reaction time, the increase of elements N and H after DAPILs treatment for different times might be due to the combination of ethanolamine and lignin in DAPILs. Many Ph-OH exist in modified lignin (PAL1), while ethanolamine contains amino (-NH_2_) and -OH groups. Under high temperature conditions, ethanolamine undergoes an amination reaction with Ph-OH in lignin to produce a small amount of aminated lignin [49,50]. This has been reported in other literature and seems to be a common feature of pretreatment of lignin with amines [51,52]. Moreover, PAL2 was observed to have more ethanolamine in comparison to that of PAL1, which could be due to the fact PAL2 was more basic with a reaction time of 6 h. Therefore, the increase in content was more significant [53]. And the degree of demethylation was calculated through the change of methoxy content. The demethylation degrees of AL by [EOA][GA] and [EOA]_2_[GA] were 59.3% and 45%, respectively.

The molecular weight of AL before and after modification was examined by GPC. As shown in Table S2, the molecular weight (M_w_) of lignin samples all increased after modification, and the increase in the M_w_ of PAL3 was particularly significant. This was possibly because PAL3 was subjected to an acidic condition, which was favorable to the condensation of lignin and led to an obvious increase in the M_w_ of the modified lignin. Moreover, it was observed that the M_w_ and M_n_ of PAL2 increased, which was due to partial condensation of lignin under a longer reaction time at 90 °C [54]. The increase in M_w_ during the modification process caused the lignin to form a highly cross-linked structure, resulting in an increase in storage modulus and greater rigidity of the lignin. Therefore, the modified lignin with higher M_w_ was commonly used to prepare resins [55].

**Figure 3:**
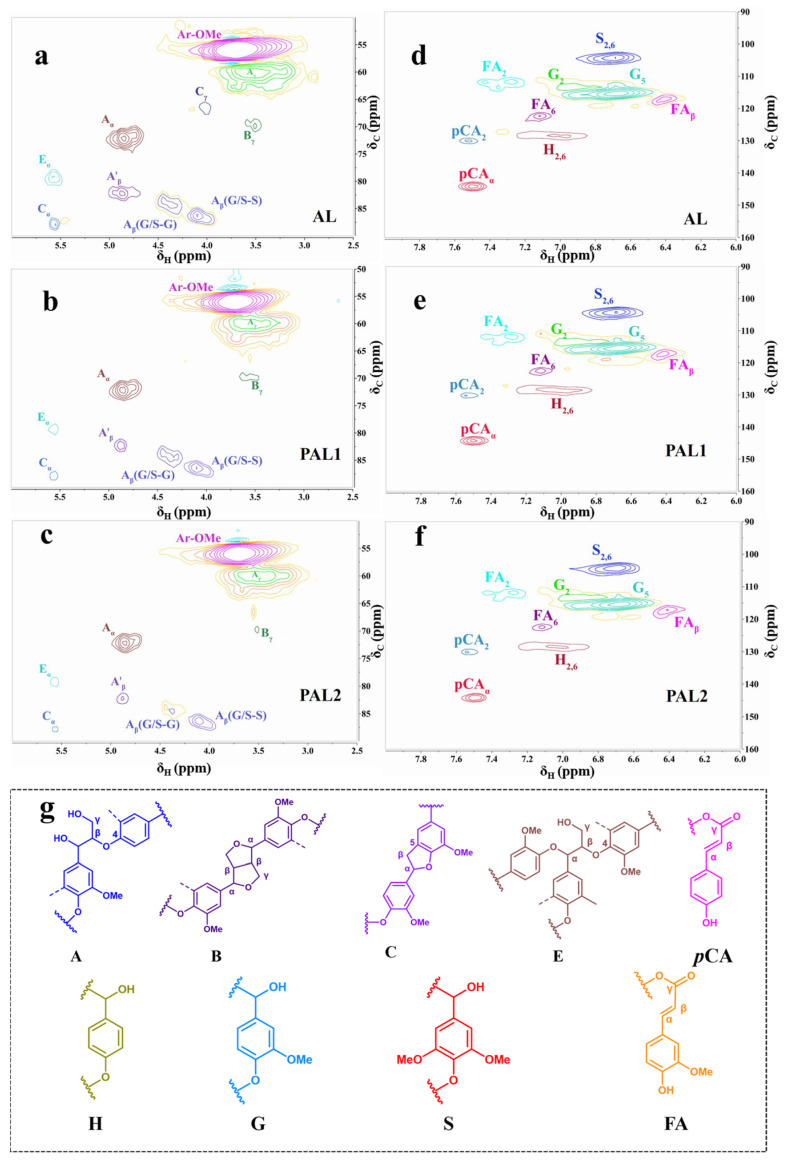
HSQC NMR spectra of lignin samples, denoted as AL, PAL1, and PAL2. (**a**–**c**) The side chain regions of AL, PAL1, and PAL2. (**d**–**f**) The aroma regions of AL, PAL1, and PAL2. (**g**) Detection of the group key structures of the lignin fractions, including changes in the units of the side chain bonds and the aromatic regions: (A) β-*O*-4′ bonds, (B) resin structure formed by β-β′ bonds, (C) phenyl formed by β-x-5′ and α-*O*-4 bonds coumarin structure, (E) α, β-diaryl ether substructures, (G) guaiacyl unit, (S) syringyl unit, (H) *p*-hydroxyphenyl unit, (*p*CA) *p*-coumarate, and (FA) ferulate.

To further investigate the mechanism of lignin demethylation and the changes of functional groups, the 2D HSQC NMR analysis was performed and the results are displayed in Figure 3 and Table 2. Table S3 is the 2D HSQC belonging table of various units in AL [56]. It is worth noting that the results of the lignin side chain region in Figure 3a–c show that the β-*O*-4′ content decreased after AL demethylation modification. In the samples of PAL1- and PAL2-modified lignin, the content of S unit and the value of S/G showed a decreasing trend (Figure 3d–f). The β-*O*-4′ content decreased from 36.14% to 23.52% and 28.91%, respectively. Moreover, the contents of A_α_ (δ_C_/δ_H_ 72.31/4.84 ppm), A_γ_ (δ_C_/δ_H_ 60.18/3.53 ppm), A_β_ (G/S-G) (δ_C_/δ_H_ 84.08/4.37 ppm), A_β_ (G/S-S) (δ_C_/δ_H_ 86.23/4.09 ppm), A’_β_ (δ_C_/δ_H_ 82.29/4.85 ppm), B_γ_ (δ_C_/δ_H_ 69.81/3.50 ppm), C_γ_ (δ_C_/δ_H_ 66.69/4.01 ppm), and E_α_ (δ_C_/δ_H_ 79.24/5.57 ppm) were found to be decreased to different degrees, and the C_γ_ peak even disappeared (Figure 3a–c). A group of representative signal strength measurement results are presented in Table S4. Those results indicated that the breaking of β-*O*-4′ structure also contributed to a fraction of low molecular weight polyphenols after the demethylation of AL (as shown in Scheme 1).

In the aromatic region of lignin, the peaks of the S, G, and H units could be observed before and after lignin modification. Notably, the peak intensities of the S and G units were found to be reduced after the modification, and the value of S/G decreased from 3.66 to 1.66. The significantly reduced peak strength of the S unit as compared to that of the G unit was due to the higher methoxyl content of the S unit, which was more prone to fracture than the G unit. In addition to the basic unit structure, the correlation signals of *p*-coumaric acid (*p*CA) and ferulic acid (FA) were observed in the aromatic region. The decrease in the intensity of *p*CA and FA in PAL1 and PAL2 in comparison with that of AL may be due to the preferential breaking of the ester bonds of *p*CA and FA during the modification process [57].

### 2.3. Molecular Computation

**Figure 4:**
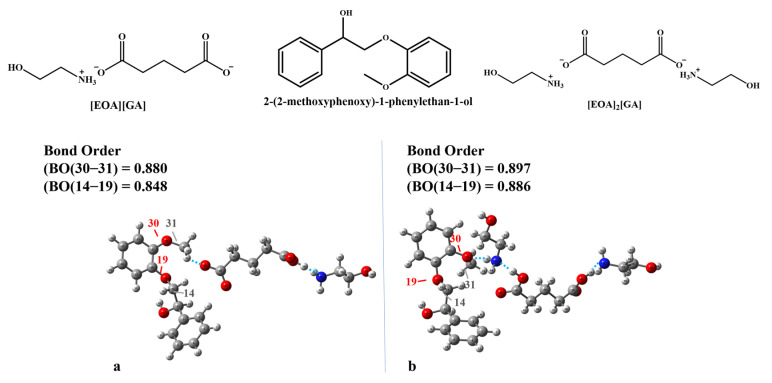
Computational simulations of the interaction between DAPIL and lignin model compounds. (**a**) [EOA][GA]-2-(2-methoxyphenoxy)-1-phenylethan-1-ol and (**b**) [EOA]_2_[GA]-2-(2-methoxyphenoxy)-1-phenylethan-1-ol.

The reactions of the lignin model compound with two different types of DAPILs at the bond level were analyzed by molecular simulations. As depicted Figure 4, the carboxyl groups (-COOH) of both ILs could interact with the lignin model compounds in the system of two bidentate ILs. According to simulations performed with the Multiwfn software (gaussian 16 A03) [58], the bond orders for the C31-O30 bond were 0.880 and 0.897, and for the C14-O19 bond were 0.848 and 0.886, depending on whether [EOA][GA] or [EOA]_2_[GA] was used. This positive correlation existed between bonding level and bonding stability. Accordingly, a positive correlation between bonding level and bonding stability was observed. The higher the bonding energy level, the more stable the bonding [59]. A high bonding level also indicated that the proton-coupled electrons were involved in the bond formation, and the density of electrons increased the gravitational attraction between each other, thus improving the bond stability. Based on the calculation results, it can be concluded that the bonding level of the lignin model compounds is lower in the [EOA][GA] simulation system, in which the carboxyl group of DAPIL has a higher density of electron cloud, which is more likely to undergo nucleophilic reaction with C31-O30, allowing the -CH_3_ to be shed and free Ph-OH to be formed. Moreover, in the [EOA][GA] system, the acidic environment catalyzed the breaking of the lignin β-*O*-4′ bond, which also increased the PHC [60]. Combined with the previously reported literature [61], we speculate that DAPIL acts as a catalytic cleavage of β-*O*-4′ bonds in addition to acting as a solvent during demethylation. This suggests that in addition to the demethylation effect of the increase in Ph-OH, the breaking of the β-*O*-4′ bond is also an important cause (Figure S4).

### 2.4. DAPIL Cycle Performance

**Figure 5:**
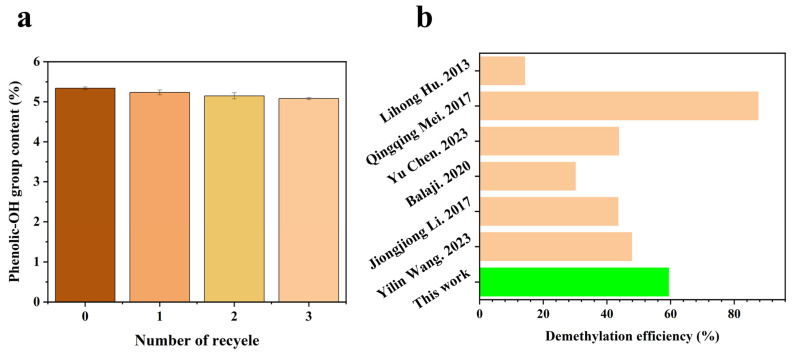
(**a**) DAPIL recycling and (**b**) demethylation efficiency of different methods. (Lihong Hu. 2013 [24], Qingqing Mei. 2017 [32], Yu Chen. 2023 [65], Balaji. 2020 [66], Jiongjiong Li. 2017 [67], Yilin Wang. 2023 [68]).

Finally, in addition to the circular performance of [EOA][GA], we also compared the demethylation efficiency of [EOA][GA] with other traditional demethylation reagents, as shown in Figure 5 and Table S5 [20,27,62,63,64,65]. As shown in Figure 5a, after the third cycle, the PCH of lignin decreased from 5.24% in the first cycle to 5.08%, a reduction of 0.16%. It was only 0.26% lower than AL after demethylation of fresh DAPIL. It also shows that it has good recyclability. When compared with the content of phenolic hydroxyl groups depicted in Figure 2a, an increase is observed, likely attributable to the inherent heterogeneity of lignin. The challenge of homogenizing lignin to enhance its high-value utilization remains a significant hurdle that demands our attention. In addition, ^1^H NMR analysis was performed on the DAPIL before and after the reaction, as shown in Figure S3. The DAPIL is recovered according to the method described in Section 2.4. The peak at 6.90 ppm is the peak of DAPIL active H, and the peaks at 3.05 ppm and 3.35 ppm are the peaks of amide. The formation of amide is the reaction of the ethanolamine and carboxyl group under the condition of heating in this experiment [66]. Most of the peaks of RDAPIL did not shift after cycling. However, due to the consumption of a small amount of ethanolamine in the generation of amide, the electron cloud density around the proton decreases, causing the peak of active hydrogen to shift to a lower area (from 6.9 ppm to 7.7 ppm). The spectra did not reveal any characteristic peaks of lignin, which also indicated that lignin was essentially separated from the DAPIL and did not require further purification. As shown in Figure 5b and Table S5, chemical methods such as pyrolysis, the biological method, and Lewis acid can demethylate lignin, split aryl ether bonds in lignin macromolecules, and replace natural methoxy groups with hydroxyl groups. The demethylation efficiency of AL in this work is about 59.3%, which is better than many currently available demethylation reagents. Although demethylation efficiency needs to be further improved, [EOA][GA] is less toxic than metal halides, in more benign conditions, and can be recycled, which is more cost-effective.

## 3. Materials and Methods

### 3.1. Materials

Alkali lignin was obtained from Shandong Longli Bio-technology Co. of China (Dezhou, China). Succinic acid was purchased from Zancheng (Tianjin, China) Technology Co. (Tianjin, China). Ethanolamine (EOA, 99%), dioxane (AR, 99%), ethanol (CH_3_CH_2_OH, 99%), sodium hydroxide (NaOH, 99%), succinic acid (SA, 99%), glutaric acid (GA, 99%), L-aspartic acid (Asp, 99%), and L-glutamic acid (Glu, 99%) were obtained from Shanghai Meryer Biochemical Technology Co. (Shanghai, China). Hydrochloric acid (HCl, 37%) was purchased from Shanghai McLean Biochemical Technology Co. (Shanghai, China). All raw materials are used directly, except for lignin, which requires further purification.

### 3.2. Lignin Purification Treatment

In a typical procedure, an appropriate amount of crude lignin was dispersed in a 30 wt% NaOH solution and stirred for 2 h. Then vacuum filtration was carried out using 10 cm of qualitative medium-speed filter paper (Newstar, Hangzhou Special Paper Industry Co., Ltd., Hangzhou, China), the supernatant was adjusted to pH = 2 with 6 M HCl and continuously stirred for 3 h at 50 °C. Finally, the mixed system was centrifuged at 8000 rpm for 5 min. The lignin precipitate was separated by centrifugation, and the purified lignin was obtained and recorded as AL.

### 3.3. Preparation of DAPILs

To synthesize the corresponding 8 DAPILs, the dibasic acid (i.e., Asp, Glu, SA, and GA) and organic base (EOA) were mixed according to a certain stoichiometric ratio with the ratios of base to acid in the range of 1:1 to 2:1. In a typical preparation, 6.655 g of aspartic acid was added to 6.1083 g of ethanolamine with 5 mL of deionized water and stirred at 80 °C until the solution was clear to equilibrate proton exchange.

### 3.4. Demethylation of Purified Lignin in ILs and Recovery of DAPILs

The water content in ILs significantly impacts lignin solubility. Based on previous literature, we add an appropriate amount of deionized water to the synthesized viscous ionic liquid to lower its viscosity [67,68]. In total, 0.5 g AL, 2 g IL, and 0.5 mL deionized water were added into a 15 mL sealed high-pressure glass tube and heated at different temperatures (70, 90, and 110 °C) for 2 h. After the reaction, the system was cooled to room temperature. Lignin dissolved in DAPILs was precipitated using an anti-solvent method. Specifically, 5 g of ethanol was added to the system, followed by an operation of ultrasonic vibration for 5 min. Then, excess water was added to precipitate the modified lignin from the system. When the suspension had been transferred to a centrifuge tube, it was conducted by rotating at 8000 rpm for 5 min to separate the lignin from the DAPILs. The lignin was centrifuged and washed with 10 mL of deionized water. This was repeated three times. The separated lignin was freeze-dried at −81 °C and 10 Pa for 12 h. The DAPIL in the washing solution containing DAPIL was recovered by using a rotary evaporator (60 °C, 2 h). The recovered DAPIL was denoted as RDAPIL.

### 3.5. Measurement of Ph-OH in Lignin Species by Ultraviolet Differential Photometry

According to the method reported in the literature [69,70,71], the solution with pH 6 and 0.2 mol/L NaOH was firstly prepared. Specifically, a standard solution with a pH of 6 (solution 1) was made by volumes of 3.368 g of KH_2_PO_4_ and 0.113 g of NaOH in a 500 mL volumetric flask. Then, a 0.2 mol/L NaOH solution (solution 2) was prepared by adding 4 g NaOH into 500 mL DI water. A total of 5~10 mg of modified lignin was dissolved in 10 mL of aqueous dioxane (9:1, dioxane/water) (solution 3). Two 50 mL volumetric flasks were taken and 2 mL of solution 3 was added to each of them and then fixed to scale with solution 1 and solution 2, respectively. The instrument used was the ultraviolet-visible spectrophotometer (Hitachi U-3900, Hitachi High—Tech Science Corporation, Shizuoka, Japan), and the cuvette path length (L) was 1 cm. With solution 1 as the reference, the absorbance curve of solution 2 in the range of 200~400 nm was measured. The phenolic hydroxyl content (PHC) of lignin was calculated by the peak intensity at 300 nm and 360 nm in the absorbance curve. The PHC calculation formula is as follows:

(1) $$\mathrm{PHC}=\frac{250\;\times \;(A_{1}\;\times \;0.182+A_{2}\;\times \;0.425)\;}{m_{s}\;\times \;L}$$

In the above Equation (1), *A*_1_ represents the intensity of the absorption peak near 360 nm, *A*_2_ represents the intensity of the absorption peak near 300 nm, *m_s_* represents the mass of the weighed sample in mg, L denotes path length in cm.

### 3.6. Characterization of Lignin by Fourier Transform Infrared Spectroscopy

Fourier transform infrared spectra (FTIR) (IRTracer-100, Shimadzu, Kyoto, Japan) were scanned with the frequency region of 4000~400 cm^−1^. The lignin was characterized by FTIR using the KBr pellet method. The KBr powder and lignin powder were mixed and ground (100/1, m_KBr_/m_lignin_), pressed into tablets for infrared characteristic peak characterization, and parallel measurements were made three times.

### 3.7. Gel Permeation Chromatography (GPC) Analysis of Lignin

GPC was performed using Waters 1515 GPC (Waters Corporation, Milford, MA, USA). Firstly, the raw lignin sample was acetylated to increase its solubility. Typically, 100 mg of lignin, 2 mL of pyridine and 2 mL of acetic anhydride were placed in a 15 mL vial and stirred in a dark environment for 24 h. At the end of the reaction, to maintain structural integrity, the acetylated lignin was precipitated using DI water as an anti-solvent.

Before the test, the Waters 1515 GPC system (GPC Breeze System; Milford, MA, USA) was calibrated using the universal calibration method. After that, 4 mg acetylated lignin was dissolved in 8 mL THF at a mobile phase elution rate of 1 mL/min.

### 3.8. Elemental Analysis

Lignin was analyzed for elements using an organic elemental analyzer (Leeman EA3000, LEEMAN LABS INC, Hudson, NH, USA). A totla of 30 mg of lignin was taken for C, H, and N contents and O content was obtained by difference calculation.

### 3.9. ^1^H NMR of Lignin and DAPILs

The lignin samples were acetylated to better dissolve the lignin in the system, and the strong solvent DMSO-d_6_ was used as the system solvent. ^1^H NMR samples were prepared by weighing appropriate amounts of acetylated lignin and *p*-nitrobenzaldehyde (NBA) (4/1, w_Lignin_/w_NBA_) dissolved in DMSO-*d*_6_. The experimental tests were performed using a Bruker 400 MHz with 128 scans at room temperature, and the relative content of the specific functional groups in the lignin samples was calculated according to the formula. The specific formula for calculating the relative content of functional groups is given below:

(2) $$F=(M_{i}/I_{i}\times A)/M\times 100\%$$

In the above equation, *F*: relative content of specific functional groups (mmol/g); *M_i_*: weight of the inner label NBA (mg); *I_i_*: peak area of internal standard NBA; *A*: peak area of functional groups; *M*: weight of lignin samples.

### 3.10. 2D HSQC NMR

2D Heteronuclear single quantum coherence (HSQC) NMR was performed on lignin ^1^H NMR and ^13^C NMR spectra using BRUKER AVANCE (Bruker Corporation, Karlsruhe, Germany) 400 at 25 °C. A total of 500 mg of lignin sample was dissolved in 500 μL of DMSO-*d*_6_. To obtain more accurate results, the experimental parameters should be set as follows: spectral widths of 5000 Hz for HSQC on ^1^H NMR and 18,000 Hz on ^13^C NMR, respectively. The DS and NS were 16 and 110, respectively, and a relaxation delay of 2.0 s. The contents of different types of lignin (G, S, and H) were determined by a semiquantitative computational method, and the plots were based on the center peak of DMSO-*d*_6_ (δ_C_/δ_H_ at 39.5 ppm/2.49 ppm) as an internal reference.

### 3.11. DAPILs Conductivity Test

Measure the conductivity of DAPILs with a conductivity meter (Shanghai Remco DDSJ-308F, Shanghai, China). A standard solution of conductivity (1408 μs/cm) was first prepared. A total of 0.744g KCl was put into a 1000 mL volumetric flask, and then fixed to the scale, which was used as the standard solution. The conductivity of eight DAPILs was then determined.

### 3.12. Determination of the pH Value of DAPILs

pH measurements of the DAPIL were performed using a pH meter (LeiCi PHS-3C, Shanghai Yidian Science Instrument Co., Ltd., Shanghai, China). Prior to use, the electrode was rinsed with deionized water and gently dried with filter paper. Calibration was carried out using standard buffer solutions at pH 6.86 and pH 4.00. After calibration, the electrode was cleaned again and immersed into the DAPIL sample. After the reading stabilizes, record the pH value, repeat twice, and calculate the average.

### 3.13. Meyer Bond Orders

The Gaussian 16 suite of programs was used to optimize the configurations with the B3LYP functional and the 6-311G (d,p) basis set [72]. Meyer bond orders of all compounds were analyzed using Multiwfn software (gaussian 16 A03) [58].

## 4. Conclusions

In summary, a DAPIL [EOA][GA] with dual active sites was developed in this paper. Through the demethylation of the DAPIL system, the demethylation efficiency reached 59.3%, and the lignin polyphenol content was increased by 1.58 times. Through a series of characterization methods, it was proved that demethylation of lignin was accompanied by effective cleavage of β-*O*-4′ bond, and both ways increased the content of the phenol hydroxyl group. This method is simple to synthesize, has a short reaction time and mild reaction conditions, and further verifies the feasibility of customized ILs system-modified lignin. DAPIL-modified lignin is a promising method for lignin modification. It not only broadens the application field of DAPILs, but also provides a new way for the appreciation of lignin. The improvement of lignin polyphenol properties has great application potential in the pharmaceutical, chemical, construction, and other fields.

## Figures and Tables

**Table 1:** Relative signal intensities of methoxy and hydroxy groups in ^1^H NMR.

Sample	M/mg	M_i_/mg	Chemical Shift			F_Ar-OH_ (mmol/g)	F_MeO_ (mmol/g)
			I_i_ δ_10.3–10.1_	I_ar-OH_ δ_2.46–2.11_	I_MeO_ δ_4.0–3.71_		
AL	40	5	1	4.05	8.86	0.50	1.11
PAL1	40	5	1	4.68	3.64	0.58	0.45
PAL2	40	5	1	4.93	5.93	0.61	0.61

F: relative content of specific functional groups (mmol/g). M_i_: weight of the inner label NBA (mg). I_i_: peak area of internal standard NBA. M: weight of lignin samples.

**Table 2:** 2D HSQC NMR analysis of different lignin samples.

Lignin Sample	Inter-Unit Linkages (/100 Ar_a_)			Lignin Aromatic Units (%)		S/G	
	β-*O*-4′	β-β′	β-5′	S	G	H	
AL	36.14	12.04	7.22	66.46	17.3	19.23	3.66
PAL1	23.52	9.41	4.7	44.77	26.86	28.35	1.66
PAL2	28.91	9.6	4.81	51.51	25.75	22.72	2

## Data Availability

Data are contained within the article and Supplementary Materials.

## Supplementary Materials

The following supporting information can be downloaded at: https://www.mdpi.com/article/10.3390/molecules30112445/s1.
