# Supplementary material for: Finely Designing Dicarboxylic Acid-Based Protic Ionic Liquids System for Tailoring Lignin Structure via Demethylation Strategy
Source: Molecules. 2025 Jun 3;30(11):2445. doi: 10.3390/molecules30112445 (PMC12156136; doi:10.3390/molecules30112445)
Supplement: Supplementary file 1 [file molecules-30-02445-s001.zip › molecules-3608086-supplementary.pdf]

Supporting Information

Finely designing dicarboxylic acids based protic ionic liquids system for tailoring lignin structure via demethylation strategy

Cheng Li [a, b], Xinyu Xiao [a], Qizhen Luo [b, c], Wanting Zhao [b, c], Wenzhe Xiao [b, c], Ling-Ping Xiao *[a], Yao Tong [a], Shangru Zhai *[a, d], Jian Sun *[b, c]

[a] *Liaoning Key Lab of Lignocellulose Chemistry and BioMaterials, Liaoning Collaborative Innovation Center for Lignocellulosic Biorefinery, College of Light Industry and Chemical Engineering, Dalian Polytechnic University, Dalian 116034, PR China*

[b] *Key Laboratory of Molecular Medicine and Biotherapy in the Ministry of Industry and Information Technology, School of Life Sciences, Beijing Institute of Technology, Beijing 100081, PR China*

[c] *Beijing Engineering Research Center of Cellulose and Its Derivatives, Advanced Research Institute of Multidisciplinary Sciences, Beijing Institute of Technology, Beijing 100081, PR China*

[d] *School of Environmental and Nature Resources, Zhejiang University of Science and Technology, Hangzhou 310023, PR China*

* Corresponding authors.

E-mail: lpxiao@dlpu.edu.cn (L.-P. Xiao); zhaisrchem@163.com (S.-R. Zhai); jiansun@bit.edu.cn (J. Sun)

Catalogue:

Figure S1. Eight DAPILs.

Figure S2. Eight DAPILs structural formulas.

Figure S3. ^{1}H NMR of DAPIL and RDAPIL.

Figure S4. The dual role of DAPILs.

Table S1. Changes in elemental content before and after lignin modification.

Table S2. Molecular weight of lignin before and after modification.

Table S3. Assignment of main ^{13}C–^{1}H cross-signals in the 2D HSQC spectra of the AL.

Table S4 The representative absolute intensity and relative abundance of the internal standard and lignin structure

Table S5. Comparison of demethylation efficiency of lignin with different methods.

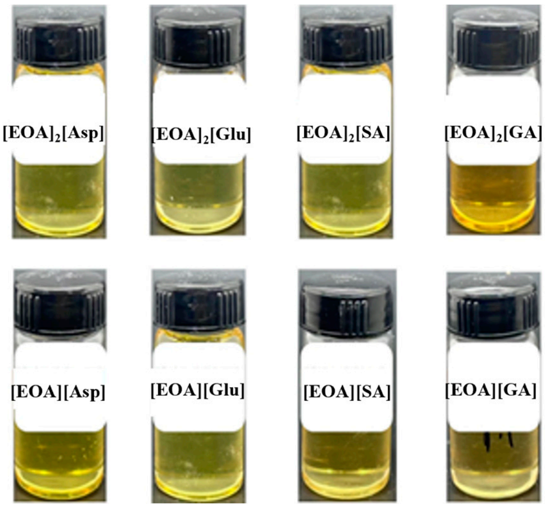

Figure S1. Eight DAPILs.

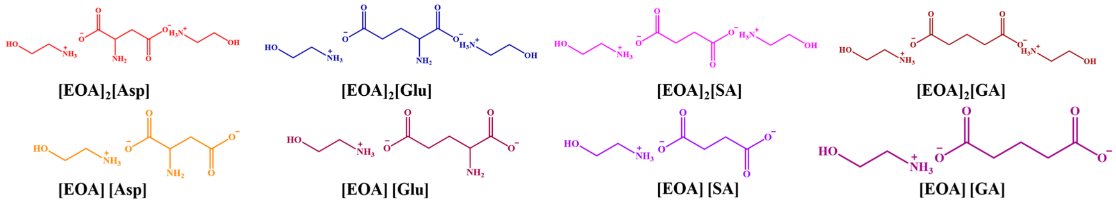

Figure S2. Eight DAPIL structural formulas.

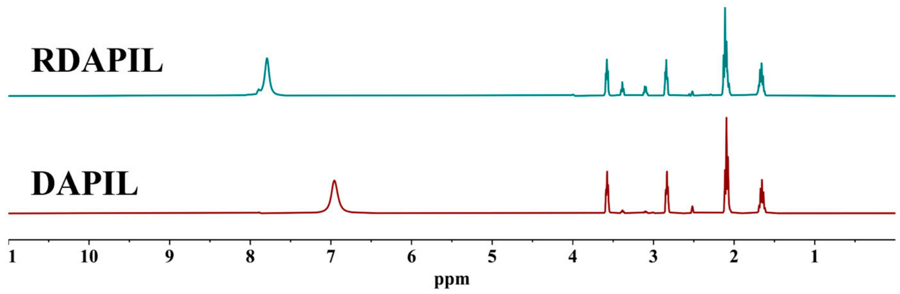

Figure S3. ^{1}H NMR of DAPIL and RDAPIL.

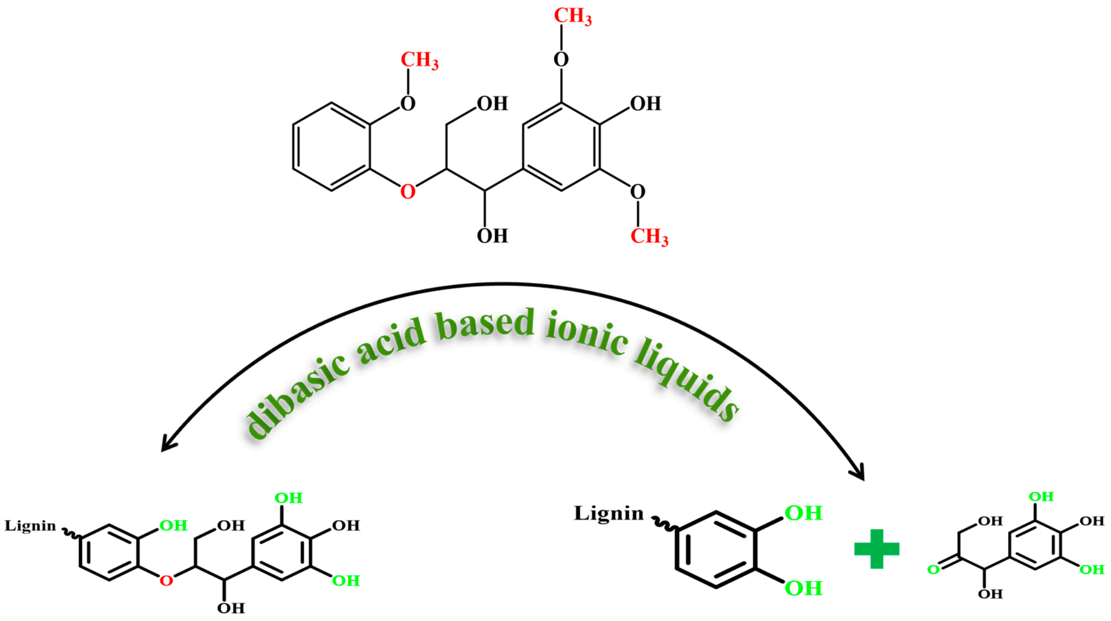

Figure S4. The dual role of DAPILs.

Table S1. Changes in elemental content before and after lignin modification

Lignin samples	C (%)	H (%)	N (%)	O (%) [a]
AL	64.033	6.274	1.332	28.361
PAL1	63.669	6.297	1.654	28.380
PAL2	63.673	6.517	1.924	27.886

[a] O contents was obtained by difference calculation.

Table S2. Molecular weight of lignin before and after modification.

Lignin samples	M_w (g/mol)	M_n (g/mol)	PDI (M_w/M_n)
AL	2151	1457	1.47
PAL1	4671	1992	2.34
PAL2	2370	1543	1.53
PAL3	6767	1497	4.52

PAL1: AL was treated in [EOA][GA] (n_{EOA}:n_{GA}=1:1) at 90 °C for 3 h.

PAL2: AL was treated in $[EOA]_2[GA]$ (n_{EOA}:n_{GA}=2:1) at 90 °C for 3 h.

PAL3: AL was treated in $[EOA][GA]_2$ (n_{EOA}:n_{GA}=1:2) at 90 °C for 3 h.

Table S3. Assignment of main ^{13}C–^{1}H cross-signals in the 2D HSQC spectra of the AL.

Lable	δ_C/δ_H (ppm)	Assignments
OCH_3	56.02/3.71	C–H in methoxyls
A_α	72.45/4.85	C_α–H_α in β–O–4 unit (A) (Erythro)
A_γ	59.97/3.55	C_γ–H_γ in β–O–4 substructures (A)
A'_β	82.14/4.86	C_β–H_β in acylated β–O–4 linked to G (A)
A_β(G/S-G)	84.00/4.39	C_β–H_β in β–O–4 linked to G (A)
A_β(G/S-S)	86.09/4.09	C_β–H_β in β–O–4 linked to S (A, Thero)
B_γ	69.76/3.50	C_γ–H_γ in β-β resinol (B)
C_α	87.85/5.56	C_α–H_α in phenylcoumaran (C)
C_γ	66.61/4.01	C_γ–H_γ in phenylcoumaran (C)
E_α	79.25/5.57	C_α–H_α in α, β-diaryl ethers (E)
$S_{2,6}$	104.56/6.68	$C_{2,6}$–$H_{2,6}$ in syringyl units (S)
G_2	113.05/6.94	C_2–H_2 in guaiacyl units (G)
G_5	115.30/6.66	C_5–H_5 in guaiacyl units (G)
$H_{2,6}$	128.49/7.01	$C_{2,6}$–$H_{2,6}$ in H units (H)
FA_2	112.09/7.33	C_2–H_2 in ferulate (p-FA)
FA_6	122.03/7.11	C_6–H_6 in ferulate (p-FA)
FA_β	117.22/6.41	C_β–H_β in ferulate (p-FA)
pCA_2	130.14/7.52	C_2–H_2 in p-coumarate (p-CA)
pCA_α	144.17/7.49	C_α–H_α in p-coumarate (p-CA)

Table S4 The representative absolute intensity and relative abundance of the internal standard and lignin structure

structures	Absolute intensity			Relative intensity		
	a	b	c	a	b	c
Internal standard	6082.0	6596.4	6543.3	1	1	1
Ar-OMe	24552.3	25203.1	25517.2	4.04	3.82	3.9
A_α	776.6	633.1	651.0	0.13	0.1	0.1
A_γ	2321.5	1926.4	2575.5	0.38	0.29	0.39
A_β(G/S-G)	316.5	258.8	253.4	0.05	0.04	0.04
A_β(G/S-S)	292.5	269.3	286.1	0.05	0.04	0.04
A'_β	145.6	122.6	106.33	0.02	0.02	0.02
B_γ	152.4	125.2	57.42	0.03	0.02	0.01
C_α	130.2	93.7	83.14	0.02	0.01	0.01
C_γ	98.4			0.02		
E_α	122.3	78.7	83.1	0.02	0.01	0.01
$S_{2,6}$	1802.0	1734.3	1864.9	0.3	0.26	0.29
G_2	490.7	466.9	458.2	0.08	0.07	0.07
G_5	4844.0	4512.3	4726.3	0.8	0.68	0.72
$H_{2,6}$	884.5	1710.8	1609.5	0.15	0.26	0.25
pCA_α	855.6	818.2	737.7	0.14	0.12	0.11
pCA_2	343.5	188.9	277.2	0.06	0.03	0.04
FA_2	720.1	677.82	735.7	0.12	0.1	0.11
FA_6	283.7	225.48	280.3	0.05	0.03	0.04
FA_β	776.64	706.7	622.2	0.13	0.11	0.1

a: The absolute intensity and relative intensity of structures in AL

b: The absolute intensity and relative intensity of structures in PAL1

c: The absolute intensity and relative intensity of structures in PAL2

Table S5. Comparison of demethylation efficiency of lignin with different methods.

Lignin	Reagent	Reaction conditions	Demethylation efficiency	Reference
Alkali lignin	[EOA][GA]	90℃, 3h	59.4%	This work
Kraft lignin	1-Dodecanethiol	NaOMe, 130 ℃, 1h	14.2%	[24]
Kraft lignin	$RhCl_3$, LiI	$HMinBF_4$, 120℃, 12h	87.5%	[32]
Hardwood lignin	NaOH/urea solution	1000 rpm, 16h	43.7%	[65]
Kraft lignin	Wood-rot fungi	28 ℃, 7 days	30.1%	[66]
Alkali lignin	Na_2SO_3	NaOH, H_2O, 90 ℃, 1 h	43.5%	[67]
Kraft lignin	ICH	DMF, 100 ℃, 12 h	47.8%	[68]
